# Supplementary material for: Well-Differentiated Papillary Mesothelioma of the Peritoneum Is Genetically Distinct from Malignant Mesothelioma
Source: Cancers (Basel). 2020 Jun 13;12(6):1568. doi: 10.3390/cancers12061568 (PMC7352777; doi:10.3390/cancers12061568)
Supplement: Supplementary file 1 [file cancers-12-01568-s001.zip › cancers-821594-suppl-Figures.pdf]

Supplementary Materials: Well-Differentiated Papillary Mesothelioma of the Peritoneum is Genetically Distinct from Malignant Mesothelioma

Raunak Shrestha Noushin Nabavi, Stanislav Volik, Shawn Anderson, Anne Haegert, Brian McConeghy, Funda Sar, Sonal Brahmbhatt, Robert Bell, Stephane Le Bihan, Yuzhuo Wang, Colin Collins and Andrew Churg

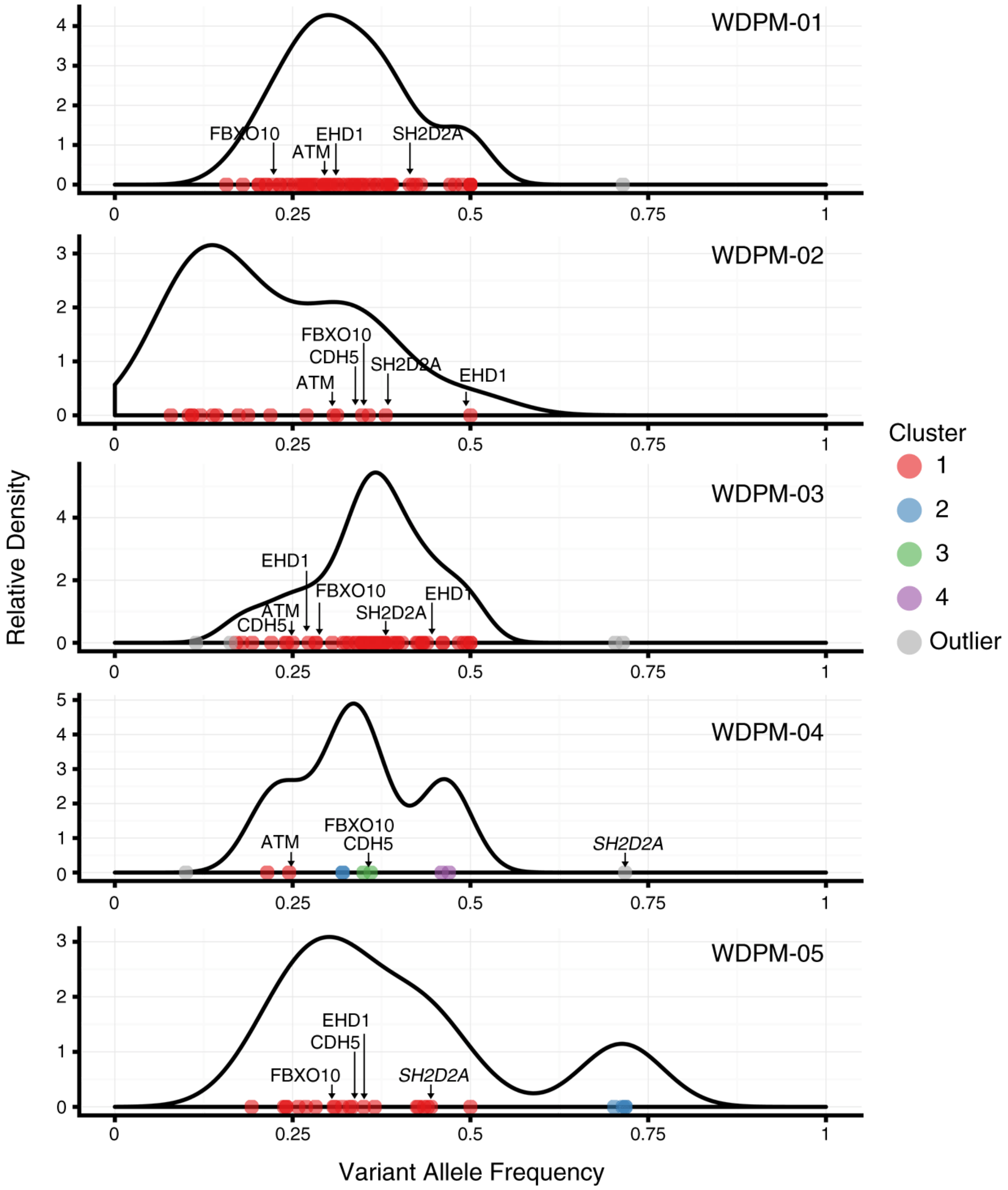

Figure S1. Distribution of variant allele frequency (VAF) in WDPM. Based on VAF, the somatic mutations identified in WDPM were clustered into different groups using the R-package Maftools.

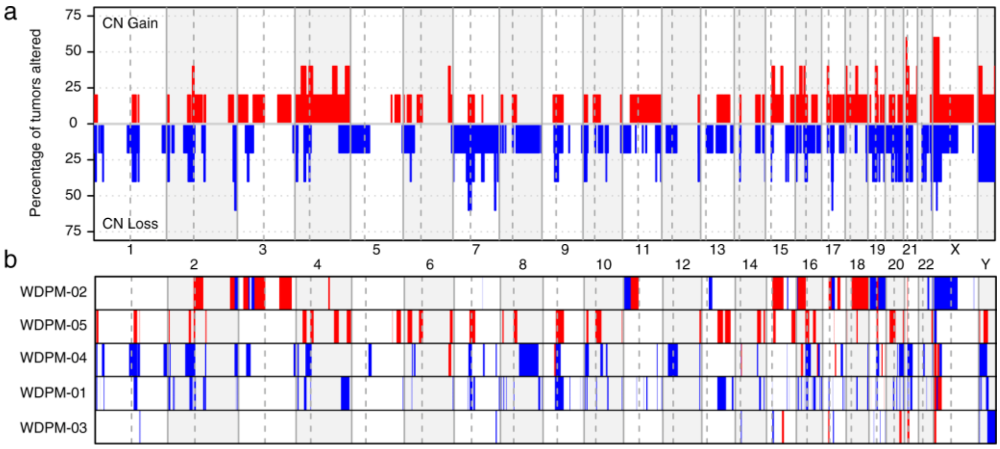

Figure S2. Landscape of copy number alterations in WDPM. The red and blue color represents the copynumber gains and copy-number loss respectively. (**a**) Aggregate copy-number alterations by chromosome regions in WDPM. (**b**) Sample-wise view of copy-number alterations. .

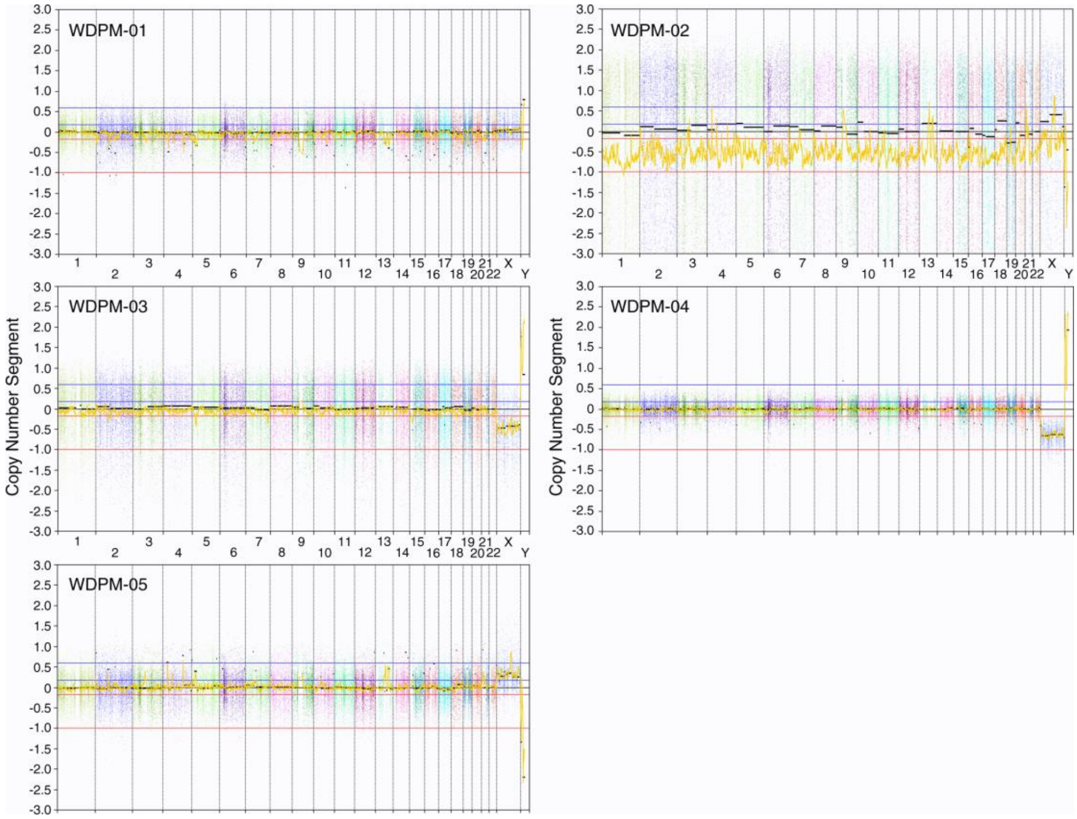

Figure S3. Copy number segments (log ratio) of WDPM samples.

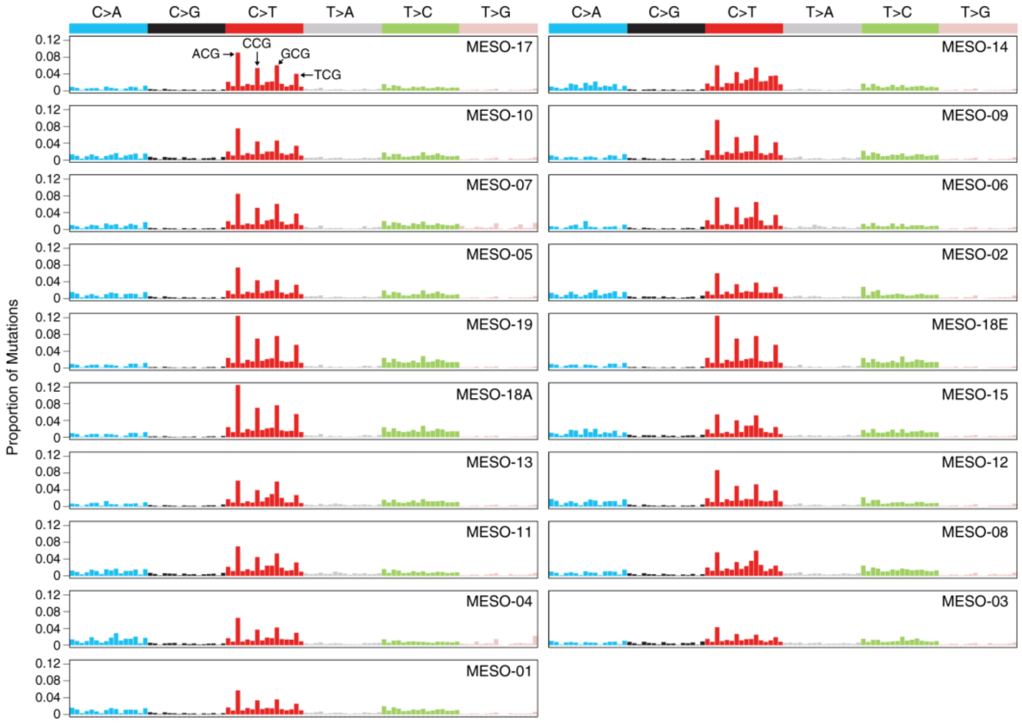

Figure S4. Mutational signature present in malignant peritoneal mesothelioma obtained from Shrestha et al, Genome Medicine 2019 [1].

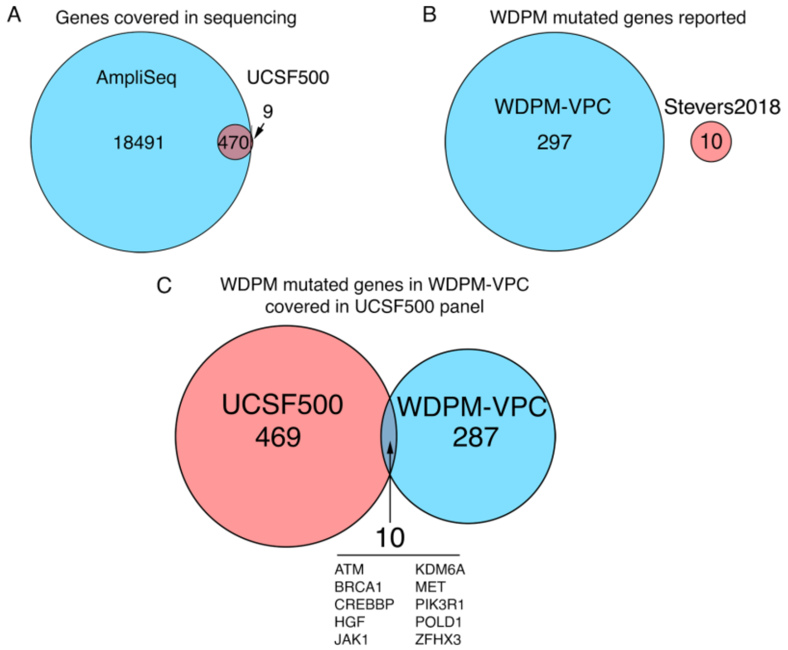

Figure S5. Comparison of Stevers et al. 2018 [2] with present study. (**A**) Venn diagram of sequencing target coverage between AmpliSeq Exome Sequencing (used our study, labelled here as WDPM-VPC) and UCSF500 gene panel (used by Stevers2018). (**B**) Venn diagram of WDPM mutated genes reported in Stevers2018 and WDPM-VPC. (**C**) Venn diagram of WDPM mutated genes in WDPM-VPC covered in UCSF500 gene panel.

Reference

1. Shrestha, R.; Nabavi, N.; Lin, Y.-Y.; Mo, F.; Anderson, S.; Volik, S.; Adomat, H.H.; Lin, D.; Xue, H.; Dong, X.; et al. BAP1 haploinsufficiency predicts a distinct immunogenic class of malignant peritoneal mesothelioma. *Genome Med.* **2019**, *11*, 8, doi:10.1186/s13073-019-0620-3.

2. Stevers, M.; Rabban, J.T.; Garg, K.; Van Ziffle, J.; Onodera, C.; Grenert, J.P.; Yeh, I.; Bastian, B.C.; Zaloudek, C.; Solomon, D.A. Well-differentiated papillary mesothelioma of the peritoneum is genetically defined by mutually exclusive mutations in TRAF7 and CDC42. *Mod. Pathol.* **2019**, *32*, 88–99, doi:10.1038/s41379-018-0127-2.

© 2020 by the authors. Licensee MDPI, Basel, Switzerland. This article is an open access article distributed under the terms and conditions of the Creative Commons Attribution (CC BY) license (http://creativecommons.org/licenses/by/4.0/).
